# The association of age at menarche and adult height with mammographic density in the International Consortium of Mammographic Density

**DOI:** 10.1186/s13058-022-01545-9

**Published:** 2022-07-14

**Authors:** Sarah V. Ward, Anya Burton, Rulla M. Tamimi, Ana Pereira, Maria Luisa Garmendia, Marina Pollan, Norman Boyd, Isabel dos-Santos-Silva, Gertraud Maskarinec, Beatriz Perez-Gomez, Celine Vachon, Hui Miao, Martín Lajous, Ruy López-Ridaura, Kimberly Bertrand, Ava Kwong, Giske Ursin, Eunjung Lee, Huiyan Ma, Sarah Vinnicombe, Sue Moss, Steve Allen, Rose Ndumia, Sudhir Vinayak, Soo-Hwang Teo, Shivaani Mariapun, Beata Peplonska, Agnieszka Bukowska-Damska, Chisato Nagata, John Hopper, Graham Giles, Vahit Ozmen, Mustafa Erkin Aribal, Joachim Schüz, Carla H. Van Gils, Johanna O. P. Wanders, Reza Sirous, Mehri Sirous, John Hipwell, Jisun Kim, Jong Won Lee, Caroline Dickens, Mikael Hartman, Kee-Seng Chia, Christopher Scott, Anna M. Chiarelli, Linda Linton, Anath Arzee Flugelman, Dorria Salem, Rasha Kamal, Valerie McCormack, Jennifer Stone

**Affiliations:** 1 School of Population and Global Health, The University of Western Australia, Perth, Australia; 2 Environment and Lifestyle Epidemiology Branch, International Agency for Research on Cancer, 150 Cours Albert Thomas, 69372 Lyon Cedex 08, France; 3 Translation Health Sciences, University of Bristol, Bristol, UK; 4 Population Health Sciences, Weill Cornell Medical College, Cornell University, New York, USA; 5 Institute of Nutrition and Food Technology, University of Chile, Santiago, Chile; 6 Cancer and Environmental Epidemiology Unit, Instituto de Salud Carlos III, Madrid, Spain; 7 Consortium for Biomedical Research in Epidemiology and Public Health (CIBERESP), Madrid, Spain; 8 Princess Margaret Cancer Centre, Toronto, ON Canada; 9 Department of Non-Communicable Disease Epidemiology, London School of Hygiene and Tropical Medicine, London, UK; 10 University of Hawaii Cancer Center, Honolulu, HI USA; 11 Department of Health Sciences Research, Mayo Clinic, Rochester, MN USA; 12 Saw Swee Hock School of Public Health, National University of Singapore, Singapore City, Singapore; 13 Instituto Nacional de Salud Pública, Cuernavaca, Mexico; 14 Slone Epidemiology Center at, Boston University, Boston, MA USA; 15 Division of Breast Surgery, Faculty of Medicine, University of Hong Kong, Pok Fu Lam, Hong Kong China; 16 Department of Surgery and Cancer Genetics Center, Hong Kong Sanatorium and Hospital, Pok Fu Lam, Hong Kong China; 17 Hong Kong Hereditary Breast Cancer Family Registry, Pok Fu Lam, Hong Kong China; 18 Cancer Registry of Norway, Oslo, Norway; 19 Department of Nutrition, Institute of Basic Medical Sciences, University of Oslo, Oslo, Norway; 20 Department of Preventive Medicine, University of Southern California, Los Angeles, CA USA; 21 Department of Population and Public Health Sciences, Keck School of Medicine, University of Southern California, Los Angeles, USA; 22 Department of Population Sciences, City of Hope National Medical Center, Duarte, CA USA; 23 Division of Cancer Research, Ninewells Hospital and Medical School, University of Dundee, Dundee, Scotland UK; 24 Wolfson Institute of Preventive Medicine, Queen Mary University of London, London, UK; 25 Department of Imaging, Royal Marsden NHS Foundation Trust, London, UK; 26 Aga Khan University Hospital, Nairobi, Kenya; 27 Breast Cancer Research Group, University Malaya Medical Centre, University Malaya, Kuala Lumpur, Malaysia; 28 Cancer Research Malaysia, Subang Jaya, Malaysia; 29 Department of Environmental Epidemiology, Nofer Institute of Occupational Medicine, Łódź, Poland; 30 Department of Physiology, Pathophysiology and Clinical Immunology,, Medical University of Lodz., Łódź, Poland; 31 Department of Epidemiology and Preventive Medicine, Graduate School of Medicine, Gifu University, Gifu, Japan; 32 Centre for Epidemiology and Biostatistics, Melbourne School of Population and Global Health, University of Melbourne, Melbourne, VIC Australia; 33 Cancer Epidemiology Centre, Cancer Council Victoria, Melbourne, VIC Australia; 34 Faculty of Medicine, Istanbul University, Istanbul, Turkey; 35 Department of Radiology, School of Medicine, Marmara University, Istanbul, Turkey; 36 Julius Center for Health Sciences and Primary Care, University Medical Center Utrecht, Utrecht, The Netherlands; 37 Radiology Department, George Washington University Hospital, Washington, DC USA; 38 Radiology Department, Isfahan University of Medical Sciences, Isfahan, Iran; 39 Centre for Medical Image Computing, University College London, London, UK; 40 Asan Medical Center, Seoul, Republic of Korea; 41 Department of Internal Medicine, Faculty of Health Sciences, University of the Witwatersrand, Johannesburg, South Africa; 42 Department of Surgery, Yong Loo Lin School of Medicine, National University of Singapore, Singapore City, Singapore; 43 NUS Graduate School for Integrative Sciences and Engineering, National University of Singapore, Singapore, Singapore; 44 Ontario Breast Screening Program, Cancer Care Ontario, Toronto, ON Canada; 45 National Cancer Control Center, Lady Davis Carmel Medical Center, Faculty of Medicine, Technion-Israel Institute Technology, Haifa, Israel; 46 Woman Imaging Unit, Radiodiagnosis Department, Kasr El Aini, Cairo University Hospitals, Cairo, Egypt

**Keywords:** Mammographic density, Menarche, Height, Breast cancer

## Abstract

**Background:**

Early age at menarche and tall stature are associated with increased breast cancer risk. We examined whether these associations were also positively associated with mammographic density, a strong marker of breast cancer risk.

**Methods:**

Participants were 10,681 breast-cancer-free women from 22 countries in the International Consortium of Mammographic Density, each with centrally assessed mammographic density and a common set of epidemiologic data. Study periods for the 27 studies ranged from 1987 to 2014. Multi-level linear regression models estimated changes in square-root per cent density (√PD) and dense area (√DA) associated with age at menarche and adult height in pooled analyses and population-specific meta-analyses. Models were adjusted for age at mammogram, body mass index, menopausal status, hormone therapy use, mammography view and type, mammographic density assessor, parity and height/age at menarche.

**Results:**

In pooled analyses, later age at menarche was associated with higher per cent density (*β*_√PD_ = 0.023 SE = 0.008, *P* = 0.003) and larger dense area (*β*_√DA_ = 0.032 SE = 0.010, *P* = 0.002). Taller women had larger dense area (*β*_√DA_ = 0.069 SE = 0.028, *P* = 0.012) and higher per cent density (*β*_√PD_ = 0.044, SE = 0.023, *P* = 0.054), although the observed effect on per cent density depended upon the adjustment used for body size. Similar overall effect estimates were observed in meta-analyses across population groups.

**Conclusions:**

In one of the largest international studies to date, later age at menarche was positively associated with mammographic density. This is in contrast to its association with breast cancer risk, providing little evidence of mediation. Increased height was also positively associated with mammographic density, particularly dense area. These results suggest a complex relationship between growth and development, mammographic density and breast cancer risk. Future studies should evaluate the potential mediation of the breast cancer effects of taller stature through absolute breast density.

**Supplementary Information:**

The online version contains supplementary material available at 10.1186/s13058-022-01545-9.

## Background

Several measures of growth and development across a woman’s life course are associated with breast cancer risk. In particular, early age at menarche and tall stature have been associated with increased breast cancer risk [1, 2], pointing to an important exposure window in early childhood and adolescence. These associations may be mediated systemically through the insulin-like growth factor (IGF) or sex steroid pathways and thereby impact on the breast parenchyma [3]. Mammographic breast density is the white radiographic appearance of epithelial and stromal tissue on a mammogram and women with increased mammographic density (MD) for their age and body mass index (BMI) are at significantly higher risk for breast cancer [4, 5]. Breast cancer and MD share common predictors, such as parity and use of hormone therapy, suggesting that the effect of these factors on breast cancer risk may be mediated, at least partly, through MD [6]. Age and BMI, a measure of weight for body size (weight/height^2^ in kilograms/metres^2^ [kg/m^2^]), are exceptions to this consistency and negatively confound the association between MD and breast cancer risk [7, 8]. That is, the true risk factor is MD adjusted for a woman’s age and BMI. There is also consistent evidence of an inverse association between pubertal body adiposity and adult MD [9–11]. Further, there is growing evidence that MD could mediate the inverse association of childhood BMI with breast cancer risk in pre-menopausal women [9, 12]. However, the associations of age at menarche and adult height, both of which are known breast cancer risk factors, with MD are less consistent and not well understood.

A recent review of pubertal mammary gland development as a determinant of adult MD summarizes the inconsistent reports of the association between age at menarche and MD. Half of the studies showed a positive association, and the other half showed either a negative or null association with MD [11]. The review also highlights the importance of adjustment for anthropometric measures when evaluating associations between age at menarche and MD, as increased body adiposity is associated with earlier pubertal development; hence, the association between age at menarche and MD is potentially dependent on childhood weight [11]. Similarly, inconsistent results have also been observed between MD and height. MD in adult women has been positively associated with both adult height [13, 14] and childhood height [15] in some studies, whilst no association has been observed in other studies [9, 16].

In the present study, we therefore examined associations of age at menarche and adult height with two measures of MD, per cent density (PD) and dense area (DA), in the International Consortium of Mammographic Density (ICMD). This international study pools data from 11,755 women from 22 countries spanning all continents worldwide, with centrally measured MD and a common core set of epidemiologic data. An important consideration when investigating the independent effects of any outcome on MD in an international study is the influence of population groups and ethnicity on observed associations. For example, age at menarche and adult stature tend to be positively correlated within populations, because an early menarche is followed by an earlier timing of the maximal height velocity and thus final adult height is shorter [17, 18]. Across populations, however, these correlation structures may differ if growth and development are associated with decreasing age at menarche and with increasing adult height [17]. These factors are taken into consideration in the present study. The diversity of ethnicities and of growth and development patterns in the ICMD enhances exposure heterogeneity and allows examination of the consistency of associations across populations.

## Methods

### Study design and participants

**Fig. 1:**
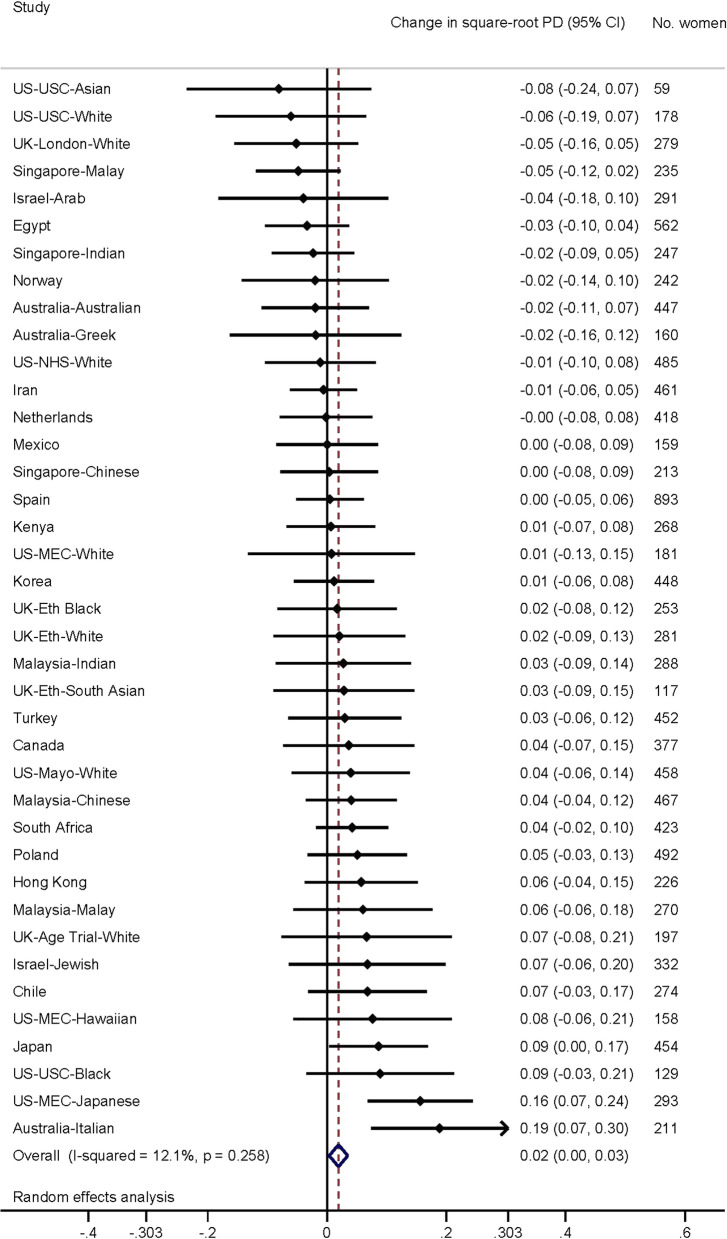
Association of age at menarche (per year) with per cent density. Forest plot depicting results from a meta-analysis of the association of age at menarche (per year) with square-root per cent density of the breast, in studies from the International Consortium on Mammographic Density. Effect estimates for each separate population group are shown, as well as the combined effect estimate, from random effects model

**Fig. 2:**
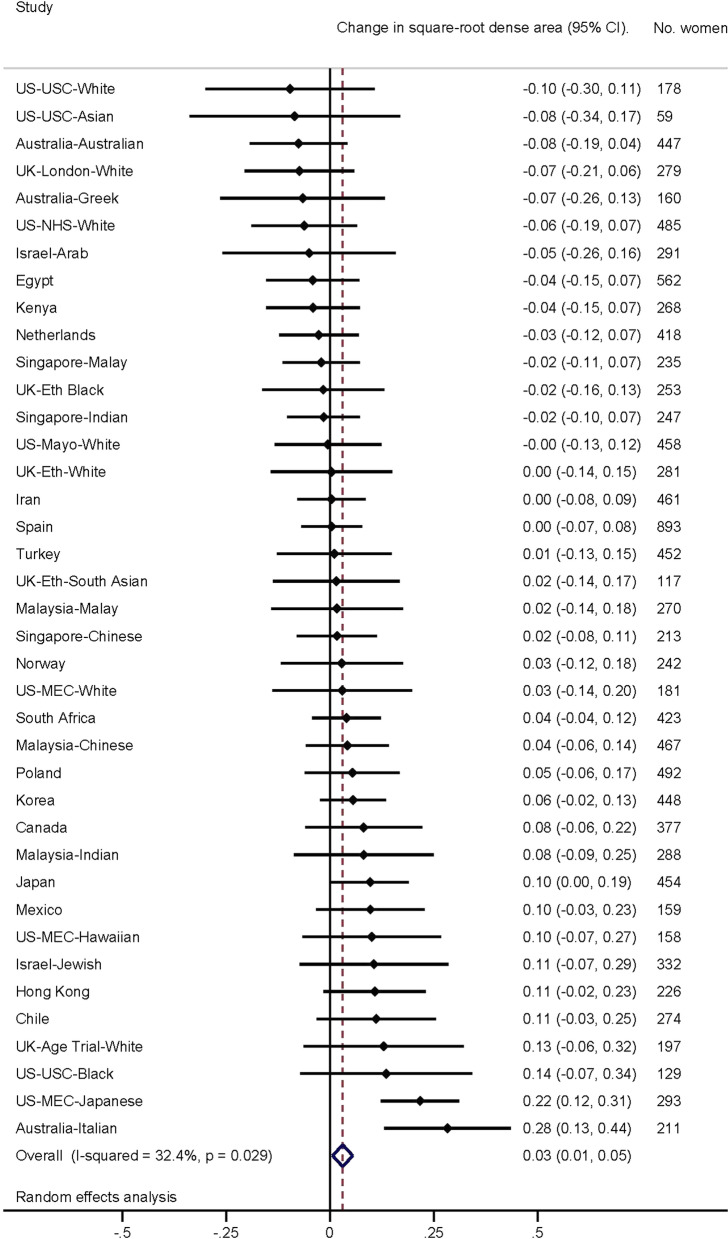
Association of age at menarche (per year) with dense area. Forest plot depicting results from a meta-analysis of the association of age at menarche (per year) with square-root dense area of the breast, in studies from the International Consortium on Mammographic Density. Effect estimates for each separate population group are shown, as well as the combined effect estimate, from random effects model

**Fig. 3:**
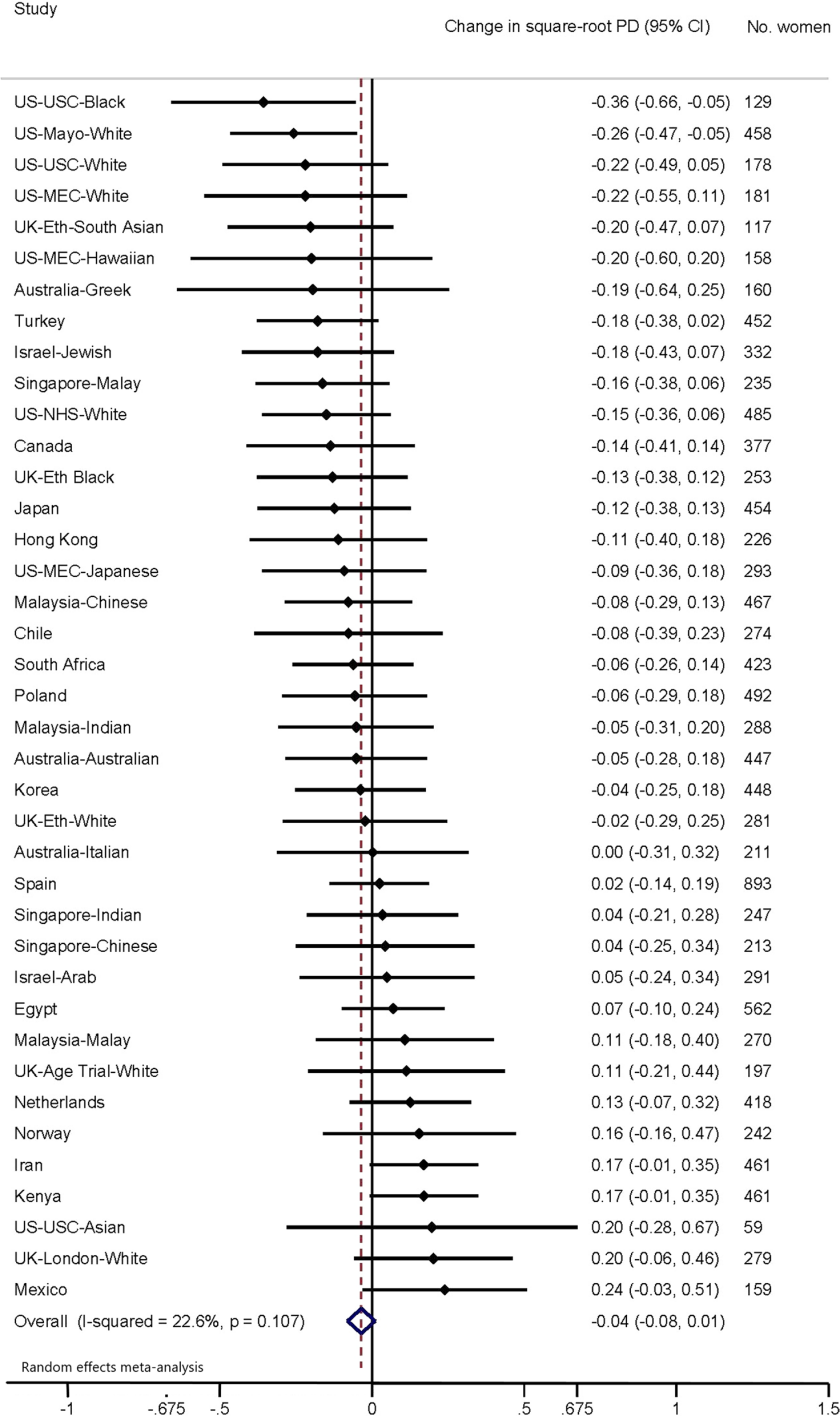
Association of adult height (per 10 cm increment) with per cent density. Forest plot depicting results from a meta-analysis of the association of adult height (per 10 cm increment) with square-root per cent density of the breast, in studies from the International Consortium on Mammographic Density. Effect estimates for each separate population group are shown, as well as the combined effect estimate, from random effects model

**Fig. 4:**
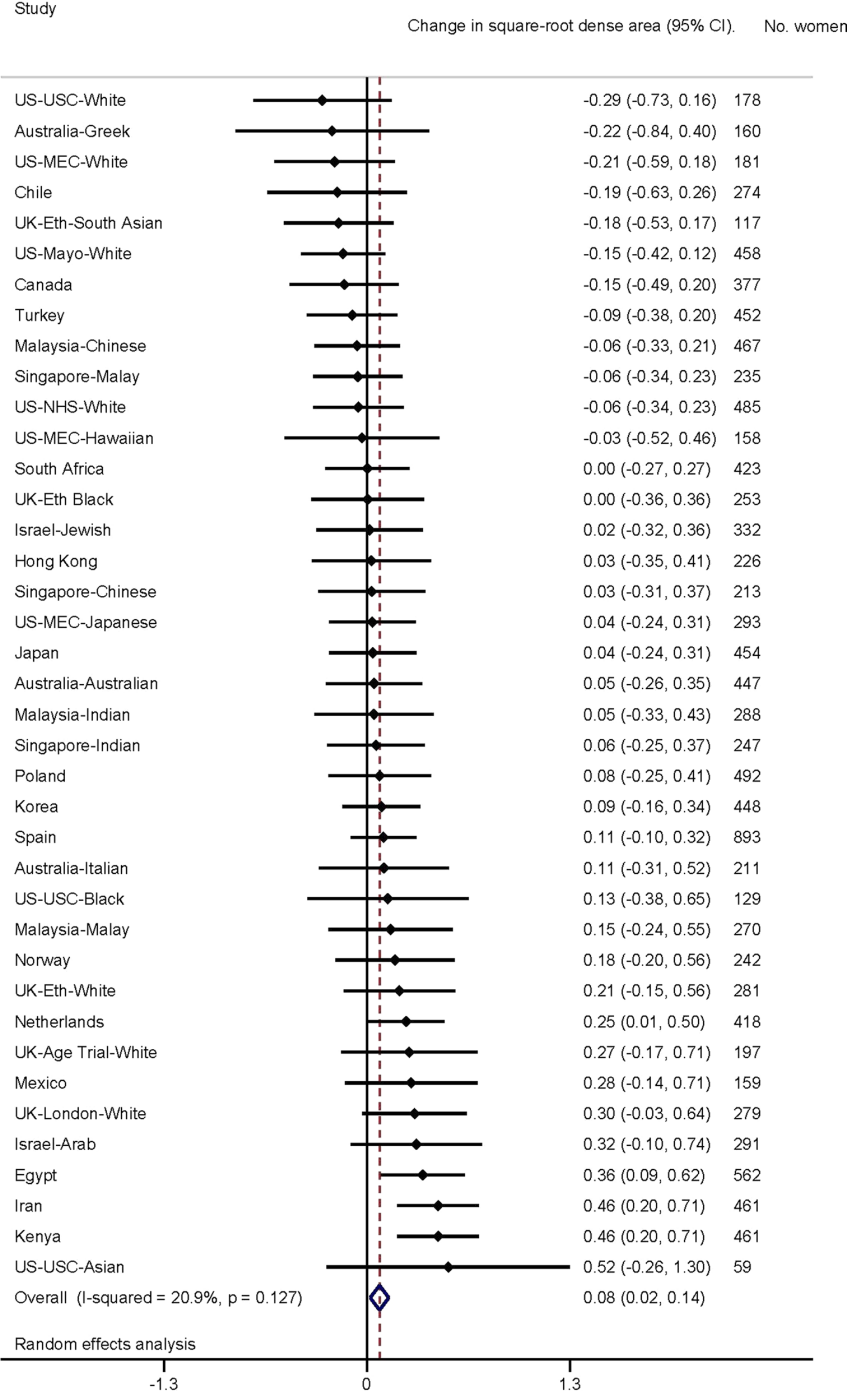
Association of adult height (per 10 cm increment) with dense area. Forest plot depicting results from a meta-analysis of the association of adult height (per 10 cm increment) with square-root dense area of the breast, in studies from the International Consortium on Mammographic Density. Effect estimates for each separate population group are shown, as well as the combined effect estimate, from random effects model

We examined two markers of developmental growth, age at menarche and adult height, in relation to measures of MD in the ICMD. The ICMD methodology and contributing studies are discussed in detail elsewhere [19]. Briefly, the consortium pooled individual-level data from studies investigating MD and its putative determinants in breast cancer-free women, purposefully including studies from diverse countries and ethnic groups with different underlying breast cancer incidence rates. In total, 11,755 women were included from 27 studies in 22 countries, forming 40 location and ethnicity-specific ‘population groups’ (Figs. 1, 2, 3, 4). Population groups included the broad ethnic groups of Black, East Asian, South Asian, Hawaiian, Mestizo, Middle Eastern and White women (see Table 1 for breakdown by country). In each population group, there were approximately 200 pre- and 200 post-menopausal women aged 35 years or older at the time of mammography. Mammograms were originally taken as part of organized screening (*n* = 13 studies), opportunistic or community-based screening (*n* = 8), mammography trials (*n* = 3) or for research (*n* = 3).

**Table 1:** Characteristics of participants in the International Consortium on Mammographic Density

World Region^a^:	All women (*n* = 10,681)	White (*n* = 4512)	Black (*n* = 938)	East Asian (*n* = 1837)	South Asian (*n* = 1133)	Mestizo (*n* = 333)	Hawaiian (*n* = 142)	Middle Eastern (*n* = 1786)
	*n* [column %] unless stated otherwise							
Per cent density (cm^2^)								
Median (IQR)	20 (11–31)	22 (16–35)	17 (10–26)	24 (16–35)	16 (8–25)	20 (13–27)	18 (9–32)	16 (8–24)
Dense area (cm^2^)								
Median (IQR)	27 (16–41)	27 (17–42)	34 (20–49)	24 (15–35)	22 (12–35)	28 (18–42)	25 (12–38)	29 (16–43)
Age at mammogram (years)								
Mean (SD)	52.7 (8.2)	53.8 (7.7)	52.4 (8.5)	52.1(8.5)	53.6 (7.2)	44.2 (6.4)	55.1 (7.5)	51.4 (8.5)
IQR	47–58	49–59	46–58	45–58	49–59	40–49	50–61	45–57
Age at menarche (yrs)^b^								
< 12.0	1470 [14]	838 [19]	80 [9]	166 [9]	153 [14]	77 [23]	20 [14]	136 [8]
12.0–12.9	2588 [24]	1031 [23]	116 [12]	437 [24]	300 [26]	87 [26]	56 [39]	561 [31]
13.0–13.9	2594 [24]	1185 [26]	167 [18]	350 [19]	283 [25]	73 [22]	0 [0]	536 [30)
14.0–14.9	2023 [19]	848 [19]	182 [19]	425 [23]	165 [15]	46 [14]	47 [33]	310 [17]
≥ 15.0	2006 [19]	610 [14]	393 [42]	459 [25]	232 [20]	50 [15]	19 [13]	243 [14]
Mean age (SD)	13.2 (1.7)	12.9 (1.6)	14.2 (2.1)	13.5 (1.8)	13.2 (1.7)	12.7 (1.7)	12.9 (1.8)	13.0 (1.4)
Height (cm)								
< 155	2760 [26]	685 [15]	186 [20]	704 [38]	663 [59]	113 [34]	17 [12]	392 [22]
155–159.9	2609 [24]	905 [20]	211 [22]	604 [33]	268 [24]	125 [38]	15 [11]	481 [27]
160–164.9	2759 [26]	1299 [29]	287 [31]	395 [22]	136 [12]	70 [21]	42 [30]	530 [30]
> 165	2553 [24]	1623 [36]	254 [27]	134 [7]	66 [6]	25 [8]	68 [48]	383 [21]
Mean height (SD)	159.4 (7.2)	162.0 (6.9)	160.6 (7.6)	156.4 (5.6)	153.2 (6.8)	157.0 (5.3)	163.2 (6.0)	159.4 (6.3)
% of measured heights^c^	66	57	39	57	85	98	0	100
BMI								
≤ 20.0	642 (6)	208 (5)	9 (1)	284 (15)	95 (8)	3 (1)	2 (1)	41 (2)
20.1–25.0	3920 (37)	1787 (40)	171 (18)	1086 (59)	376 (33)	82 (25)	43 (30)	375 (21)
25.1–30.0	2342 (32)	1503 (33)	320 (34)	411 (22)	428 (38)	133 (40)	45 (32)	592 (33)
> 30.0	2687 (25)	1014 (22)	438 (47)	56 (3)	234 (21)	115 (35)	52 (37)	778 (44)
Mean BMI (SD)	27.0 (5.7)	26.7 (5.3)	30.3 (6.2)	23.2 (3.4)	26.3 (5.0)	28.7 (5.1)	28.9 (6.3)	29.6 (6.0)
Menopausal status								
Pre-menopausal	4361 [41]	1761 [39]	328 [35]	799 [43]	324 [29]	261 [78]	35 [25]	853 [48]
Post-menopausal	6320 [59]	2751 [61]	610 [65]	1038 [57]	809 [71]	72 [22]	107 [75]	933 [52]
Ever used hormone therapy^d^								
Yes	2185 [25]	1387 [36]	133 [14]	384 [21]	82 [8]	27 [8]	78 [55]	94 [12]
No	6681 [75]	2456 [64]	802 [86]	1425 [79]	969 [92]	291 [92]	64 [45]	674 [88]
Parity								
Nulliparous	1068 [10]	661 [15]	61 [7]	161 [9]	90 [8]	21 [6]	9 [6]	65 [4]
Parous	9613 [90]	3851 [85]	877 [94]	1,676 [91]	1,043 [92]	312 [94]	133 [94]	1721 [96]
Mean parity (SD)	2.6 (1.8)	2.4 (1.1)	3.3 (1.7)	2.5 (1.2)	3.9 (2.1)	2.9 (1.4)	3.3 (1.5)	3.6 (2.2)
Age at first birth (yrs)^b^								
Mean age (SD)	24.3 (5.1)	25.3 (4.7)	22.3 (4.8)	26.2 (4.2)	22.4 (6.2)	23.9 (5.0)	22.9 (4.1)	22.5 (5.2)
IQR	21–27.5	22–27.8	19–25	23–28.3	18–26	19.8–27	19–23	19–26

*BMI* body mass index, *cm* centimetres, *IQR* inter-quartile range, *SD* standard deviation, *yrs* years

^a^Population groups and study source, by world region

White: White women in Australia (3 studies—Australian, Greek and Italian born), Canada, Netherlands, Norway, Poland, Spain, the UK (3 studies) and the USA (mainland: 3 studies, Hawaii: 1 study)

Black: Black women in Kenya, South Africa, the UK and the USA

East Asian: Chinese women in Hong Kong, Malaysia and Singapore; Japanese women in Japan, Korea and the USA (mainland: 1 study, Hawaii: 1 study)

South Asian: Malay women in Malaysia and Singapore; Indian women in India, Malaysia, Singapore and the UK

Mestizo: Mestizo women in Chile and Mexico

Hawaiian: Hawaiian women in USA (Hawaii: 1 study)

Middle Eastern: Egyptian women in Egypt; Arab women in Israel; Jewish women in Iran, Israel and Turkey

^b^Note raw data recorded categorically by some studies and subsequently converted to an approximated numerical value

^c^Height data that were measured by the original study, as opposed to self-reported

^d^Among women with non-missing data. Missing data on hormone therapy were 17% overall, < 4% for black, Hawaiian, Mestizo and East Asian, 15% missing for white women and 57% for Middle Eastern. These women were included in a missing-hormone-therapy category in later regression models

In the current study, further exclusions were made from the total 11,755 women. First, women from very small ethnic groups within each study were excluded (*n* = 129 across four countries), then women with no MD information due to poor image quality (*n* = 529), inconsistent age at first birth compared to age at menarche (*n* = 5) and ‘nulliparous’ women who had children (*n* = 6), implausible/missing BMI (*n* = 2), missing parity (*n* = 93) and missing age at menarche (*n* = 310). This resulted in a total of 10,681 women for analyses.

### Exposures of interest and confounders/modifiers

Individual-level data on sociodemographic and lifestyles factors were harmonized across all ICMD studies. Age at menarche data was self-reported in adulthood and collected as integers or categories, with the median values of each category assumed for continuous analyses. Categorical analyses were performed using previously used cut-points (< 12, 12 to < 13, 13 to < 14, 14 to < 15, and 15 years and older). Height was recorded in centimetres (cm) in the majority of studies and converted to centimetres for those recorded in feet and inches. Height was examined both as a continuous variable (per 10 cm increase) and as a categorical variable (< 155, 155 to < 160, 160 to < 165, ≥ 165 cm).

Weight was recorded in kilograms for most studies and converted from stones and pounds for all other studies. A measure of BMI was calculated for all participants as kg/m^2^. Information on the method of height and weight ascertainment was also collected, either as self-reported (*n* = 3370, 32%) or measured (*n* = 7061, 66%) and was not known in one study (*n* = 249, 2%).

Other variables included in the present analyses included age at mammogram, parity, age at first birth, and use of hormone therapy at the time of mammography. Study-specific definitions were used and then harmonized for menopausal status, as has been described in detail previously [20].

### Mammographic density measurement

In the ICMD, MD was measured centrally from digitized film mammograms and raw or processed digital images by one of three experienced assessors (authors VM, IdSS, NB) using the software program Cumulus [21]. For each woman, one mammographic image (cranio-caudal or medio-lateral oblique view) was measured. To assess the intra- and inter-assessor reliability, approximately 20% of the images were re-measured, providing repeated measures for a subset of women, as detailed previously [19]. This resulted in a total of 12,586 MD measurements for the 10,681 women in present analyses. The MD measures used in these analyses were DA (cm^2^) and PD (PD = 100 × DA/breast area), as they were considered the most aetiologically relevant. The measures from processed images were corrected to a raw image equivalent using published equations [22].

### Statistical methods

Descriptive analyses were conducted in the broad ethnic groups of Black, East Asian, South Asian, Hawaiian, Mestizo, Middle Eastern and White women to summarise the data. Two analytical approaches were then taken to examine the associations between age at menarche and adult height with the outcomes PD and DA. Both of these approaches used the more specific population groups to take into account ethnic differences between participants. Both MD outcomes were first square-root-transformed to normalize residuals. PD and DA are both area measures, so if they are considered as squares, this transformation implies that regression beta-coefficients can be interpreted as the effect on the length of the side of a square, e.g. if DA = 25 cm, √DA = 5 cm (a square of 5 × 5 cm), and beta =  + 0.1 cm, then the length of the DA square increases from 5.0 to 5.1 cm, and the corresponding DA increases from 25 to 5.1^2^ = 26.0 cm [20].

In the first analytical approach, population-specific associations were examined and their effect estimates combined using meta-analytic approaches with a random effects model. Forest plots were used to display population-specific effect estimates. Second, individual-level pooled analyses were performed using multi-level models with density measures clustered for an individual, who was clustered within their population group. Individual-level clustering was used to account for women with repeated measures, and population group clustering to account for differences in ethnicity between groups.

In both approaches, all models were adjusted for age at mammogram (cubed due to best fit), menopausal status, use of hormone therapy, mammogram view, calibration method, mammogram reader, parity and BMI (quadratic or cubed terms depending on best fit). To evaluate the possible independent effects of each exposure of interest, models for age at menarche were additionally adjusted for height and models for height were additionally adjusted for age at menarche.

Subgroup analyses by menopausal status, parity, BMI category and anthropometric ascertainment method were also performed using the multi-level pooled models. An additional adjustment for age at first birth was included for parous subsets.

### Sensitivity analyses

We also performed sensitivity analyses adjusting for a population-specific weight-for-height index, instead of BMI, as BMI and height are inversely correlated. Whilst BMI aims to be a measure of weight independent of height, it is a simplified index and is not completely independent of height [23]. The weight–height relationship has been shown to vary according to age and gender, such that height is inversely associated with BMI in (white) adults, but the magnitude of the association is larger for women and increases with age [24]. The weight–height relationship may differ in an international study where body sizes and shapes differ, and it is therefore important to examine MD associations with height independent of BMI or body fatness. Additional analyses were conducted to take these potential issues into account, using a weight-for-height index that was defined as the ratio of an individual’s weight to their population-specific expected weight. The expected weight was generated using a population group-specific relationship of weight as a function of *k*_1_ × height *k*^2^ where both *k*_1_ and *k*_2_ were optimized separately for each population group. The median value of *k*_2_ was 1.30 (inter-quartile range [IQR] 1.19–1.35; Additional file 1: Table S1), which is lower than the power of 2 which is used in BMI (i.e. weight/height^2^).

## Results

### Participant characteristics

Characteristics of the participants, overall and by ethnic group, are shown in Table 1. The mean age at mammogram was 52.7 years (standard deviation (SD) = 8.2 years), which was relatively consistent across all groups except the Mestizo women, who were, on average, approximately 10 years younger. The mean age at menarche was also similar across all ethnic groups and, on average, occurred at a 13.2 years (SD = 1.7 years). Height varied slightly across the ethnic groups, with average height notably shorter in the East Asian, South Asian and Mestizo groups. There were more post-menopausal than pre-menopausal women in all ethnic groups except the Mestizo group, consistent with their younger age, and there were substantially more parous (90%) than nulliparous (10%) women.

### Age at menarche

Forest plots depicting population group-specific associations for age at menarche with each of the MD measures, along with meta-analyses results for overall associations, are presented in Figs. 1 (PD) and 2 (DA). Overall, a small positive association was observed between the square-root change in both PD (*β* = 0.02, 95% CI 0.00, 0.03) and DA (*β* = 0.03, 95% CI 0.01, 0.05) with each yearly increase in age at menarche. Results were highly consistent across all studies for √PD (*I*^2^ = 12.1%) but less so for √DA (*I*^2^ = 32.4%), although both were considered to have low heterogeneity overall.

Pooled analyses of all participants (Table 2) showed very similar overall effect estimates to the meta-analyses. Later age at menarche was associated with increased √PD in all women (*β* = 0.057, SE = 0.008, *P* < 0.001). Results were attenuated when adjusted for BMI (*β* = 0.023, SE = 0.008, *P* = 0.003), similar to meta-analysis estimates. Adjustment for height did not alter the effect estimate substantially. Stratified analyses showed that this association was primarily driven by women with a BMI under 25 kg/m^2^ and by parous women. The association was present at both pre- and post-menopausal ages.

**Table 2:** Association of per cent density and dense area with age at menarche in pooled ICMD studies

	No. of women	No. of measurements	Per cent density^a^		Dense area^a, f^	
			Changes in square-root per cent density (SE)	P value	Changes in square-root dense area (SE)	P value
Age at menarche category (years)						
< 12.0	1470	1722	0		0	
12.0–12.9	2588	3036	0.006 (0.042)	0.889	− 0.032 (0.056)	0.575
13.0–13.9	2594	3067	0.064 (0.042)	0.130	0.053 (0.056)	0.346
14.0–14.9	2023	2397	0.057 (0.045)	0.207	0.034 (0.059)	0.569
≥ 15.0	2006	2364	0.128 (0.046)	0.005	0.166 (0.061)	0.007
*Linear associations with a 1 year increase in age at menarche*						
All women—Model A^c^						
All, BMI and height adjusted	10,681	12,586	0.023 (0.008)	0.003	0.032 (0.010)	0.002
All, without BMI adjustment	10,681	12,586	0.057 (0.008)	< 0.001	0.036 (0.010)	0.001
All, without height adjustment	10,681	12,586	0.022 (0.008)	0.004	0.033 (0.010)	0.001
Subset by BMI group (kg/m^2^)						
≤ 20.0	652	752	0.037 (0.032)	0.251		N/A^b^
20.1–25.0	3915	4585	0.039 (0.013)	0.002	0.068 (0.014)	< 0.001
25.1–30.0	3427	4048	0.013 (0.014)	0.328	0.005 (0.018)	0.782
> 30.0	2687	3201	0.012 (0.016)	0.456	0.009 (0.023)	0.704
Pre-menopausal women—Model B^d^						
All	4349	5135	0.020 (0.013)	0.122	0.028 (0.017)	0.111
Subset by parity						
Nulliparous women	483	558	− 0.044 (0.036)	0.226	0.009 (0.048)	0.847
Parous women	3866	4577	0.027 (0.013)	0.045	0.026 (0.018)	0.162
Parous women + age 1st birth adjustment	3866	4577	0.027 (0.013)	0.044	0.025 (0.018)	0.165
Post-menopausal women—Model C^d^						
All	5868	6902	0.022 (0.010)	0.029	0.029 (0.013)	0.029
All + age at menopause adjustment	5868	6902	0.022 (0.010)	0.031	0.029 (0.013)	0.031
Subset by parity						
Nulliparous women	535	626	0.005 (0.035)	0.891	0.022 (0.044)	0.623
Parous women	5333	6276	0.023 (0.011)	0.029	0.029 (0.014)	0.040
Parous women + age at 1st birth adjustment	5333	6276	0.021 (0.011)	0.051	0.026 (0.014)	0.064
Parous women + age at menopause adjustment	5333	6276	0.023 (0.011)	0.030	0.029 (0.014)	0.041
Parous women + ages at 1st birth + age at menopause adjustments	5333	6276	0.021 (0.011)	0.049	0.026 (0.014)	0.063
Nulliparous women—Model D^e^						
All	1068	1238	− 0.016 (0.025)	0.517	0.013 (0.033)	0.696
Parous women—Model E^c^						
All	9561	11,283	0.027 (0.008)	0.001	0.033 (0.011)	0.002
All + age at 1st birth adjustment	9561	11,283	0.026 (0.008)	0.001	0.033 (0.011)	0.003

*BMI* body mass index, *ICMD* International Consortium of Mammographic Density, *SE* standard error

^a^A multi-level linear regression model was used to estimate the difference in square-root per cent density and dense area associated with each per year increase in age at menarche. Models specified correlations of women (level 1) within population groups (level 2)

^b^BMI ≤ 20 category combined with BMI 20.1–25.0 categories due to lack of convergence

^**c**^Models A (all women) and E (parous women): adjusted for age at mammogram^3^, menopausal status, age at mammogram*menopausal status interaction term, BMI^3^, use of HRT, mammogram view, calibration, reader, parity, height

^d^Models B (pre-menopausal women) and C (post-menopausal women): adjusted for age at mammogram^3^, BMI^3^, use of HRT, mammogram view, calibration, reader, parity, height

^e^Model D (nulliparous women): adjusted for age at mammogram^3^, menopausal status, age at mammogram* menopausal status interaction term, BMI^3^, use of HRT, mammogram view, calibration, reader, height

^f^Dense area models: BMI^2^ adjusted for instead of BMI^3^

Similar results were observed for DA; later age at menarche was also associated with an increase in √DA for all women (*β* = 0.032, SE = 0.010, *P* = 0.002). The association with √DA was also more evident in parous women and women with lower BMI (BMI ≤ 25: *P* < 0.001) and did not differ by menopausal status. Results were generally unaffected after adjusting for age at first birth or age at menopause.

### Height

Population group-specific and meta-analyses results for the association of MD measures with height are shown in Figs. 3 (PD) and 4 (DA). Overall, there was no strong evidence of an association between √PD and height across studies (*β* = − 0.04 per 10 cm height increment, 95% CI − 0.08, 0.01), but there was evidence of an overall increase for √DA per 10 cm increase in height (*β* = 0.08, 95% CI 0.02, 0.14). Heterogeneity across population groups was relatively low for each MD measure (√PD, *I*^2^ = 22.6%; √DA, *I*^2^ = 20.9%).

Pooled results for both measures of MD and their association with adult height are presented in Table 3. For height and PD, the association was positive without adjustment for BMI or when adjusted for the study-specific weight-for-height index (Additional file 1: Table S2), but inversely associated when adjusted for BMI. The latter appeared to reflect the strong inverse association of height and BMI in almost all population groups (Additional file 1: Table S1). In the stratified analyses (adjusted for BMI), the height-√PD associations were largely negative, regardless of parity or menopausal status. When subset by the method of height measurement, an association was only observed in those women who had self-reported their height.

**Table 3:** Association of per cent density and dense area with adult height in pooled ICMD studies

	No. of women	No. of measurements	Per cent density^a^		Dense area^a,e^	
			Changes in square-root per cent density (SE)	*P* value	Changes in square-root dense area (SE)	*P* value
Height (categorical) (cm)						
< 155	2718	3240	0		0	
155–159.9	2551	3027	− 0.041 (0.037)	0.259	0.048 (0.048)	0.319
160–164.9	2683	3151	− 0.056 (0.038)	0.141	0.096 (0.050)	0.057
≥ 165	2480	2900	− 0.075 (0.042)	0.076	0.118 (0.055)	0.034
*Linear associations with a 10 cm increase in adult height*						
All women—Model A^b^						
All, without BMI adjustment	10,432	12,318	0.044 (0.023)	0.054	0.069 (0.028)	0.012
All, without age at menarche adjustment	10,432	12,318	− 0.057 (0.021)	0.007	0.062 (0.028)	0.025
All, with BMI and menarche adjustment	10,432	12,318	− 0.060 (0.021)	0.005	0.059 (0.028)	0.034
By BMI strata: (kg/m^2^)						
≤ 20.0	652	752	0.042 (0.075)	0.569	0.184 (0.073)	0.011
20.1–25.0	3882	4551	− 0.008 (0.034)	0.811	0.112 (0.041)	0.006
25.1–30.0	3328	3937	− 0.043 (0.037)	0.241	0.121 (0.050)	0.015
> 30.0	2570	3078	− 0.138 (0.040)	0.001	− 0.026 (0.060)	0.670
Subset by height measurement method						
Measured	7062	8389	− 0.040 (0.026)	0.125	0.084 (0.034)	0.015
Self-reported	3370	3929	− 0.096 (0.036)	0.007	0.020 (0.047)	0.676
Pre-menopausal women—Model B^c^						
All	4221	5000	− 0.049 (0.032)	0.130	0.074 (0.044)	0.092
Subset by parity						
Nulliparous women	478	553	− 0.201 (0.089)	0.024	− 0.012 (0.117)	0.922
Parous women	3743	4447	− 0.023 (0.034)	0.503	0.099 (0.047)	0.034
Parous women + age 1st birth adjustment	3743	4447	− 0.024 (0.034)	0.490	0.099 (0.047)	0.033
Post-menopausal women—Model C^c^						
All	5747	6769	− 0.067 (0.028)	0.017	0.047 (0.036)	0.201
All + age at menopause adjustment	5747	6769	− 0.070 (0.028)	0.013	0.043 (0.036)	0.239
Subset by parity						
Nulliparous women	531	622	− 0.144 (0.081)	0.076	− 0.084 (0.098)	0.389
Parous women	5216	6147	− 0.041 (0.029)	0.163	0.090 (0.039)	0.019
Parous women + age at 1st birth adjustment	5216	6147	− 0.045 (0.029)	0.121	0.085 (0.038)	0.027
Parous women + age at menopause adjustment	5216	6147	− 0.043 (0.029)	0.139	0.087 (0.038)	0.023
Parous women + age at 1st birth + age at menopause adjustments	5216	6147	− 0.047 (0.029)	0.107	0.083 (0.038)	0.031
Nulliparous women—Model D^d^						
All	1059	1229	− 0.197 (0.060)	0.001	− 0.043 (0.075)	0.563
Parous women—Model E^b^						
All	9321	11,024	− 0.036 (0.022)	0.111	0.090 (0.030)	0.002
All + age at 1st birth adjustment	9321	11,024	− 0.038 (0.022)	0.090	0.088 (0.030)	0.003

*BMI* body mass index, *ICMD* International Consortium of Mammographic Density, *SE* standard error

^a^A multi-level linear regression model was used to estimate the difference in square-root per cent density and dense area associated with each 10 cm increase in height. Models specified correlations of women (level 1) within population groups (level 2)

^b^Models A (all women) and E (parous women): adjusted for age at mammogram^3^, menopausal status, age at mammogram* menopausal status interaction term, BMI^3^, use of HRT, mammogram view, calibration, reader, parity, age at menarche

^c^Models B (pre-menopausal women) and C (post-menopausal women): adjusted for age at mammogram^3^, BMI^3^, use of HRT, mammogram view, calibration, reader, parity, age at menarche

^d^Model D (nulliparous women): adjusted for age at mammogram^3^, menopausal status, age at mammogram* menopausal status interaction term, BMI^3^, use of HRT, mammogram view, calibration, reader, age at menarche

^e^Dense area models: BMI^2^ adjusted for instead of BMI^3^

Consistent with the meta-analysis, an increase was observed in √DA with increased height (*β* = 0.059, SE = 0.028, *P* = 0.034) and this association was slightly stronger without adjustment for BMI (*β* = 0.069, SE = 0.028, *P* = 0.012). When stratified by BMI category, women in all but the highest (> 30 kg/m^2^) categories had an increased √DA with increased height. When analysed by height ascertainment method, the height-√DA association was stronger when height was measured (*β* = 0.084, SE = 0.034, *P* = 0.015) as opposed to self-reported (*β* = 0.020, SE = 0.047, *P* = 0.676). The association with √DA was stronger in parous women, overall (*β* = 0.090, SE = 0.030, *P* = 0.002), and in both pre- (*β* = 0.099, SE = 0.047, *P* = 0.034) and post-menopausal parous women (*β* = 0.090, SE = 0.039, *P* = 0.019).

No differences in results were observed for any outcome measures when adjusted for age at first birth in parous women or age at menopause in the post-menopausal group.

## Discussion

Within one of largest international MD studies consisting of populations with different ethnic backgrounds, we found that later age at menarche was positively associated with both per cent and absolute dense area and that increased height was positively associated with absolute dense area. Thus, the protective effect of later age at menarche on breast cancer risk is not likely mediated through MD. However, the increased risk of breast cancer associated with height could be mediated through MD, particularly DA. These results are consistent with previous findings [25] but are the first to demonstrate these associations across 22 different countries representing at least seven broad ethnic groups.

Earlier menarche is an established risk factor for breast cancer, perhaps explained by an increased number of regular menstrual cycles over the lifetime [1, 26]. As the relative amounts of epithelial, stromal and adipose tissue determine the radiological appearance of the adult breast, puberty is likely a key developmental stage in the establishment of MD [11]. As increased MD is associated with increased breast cancer risk, the paradoxical positive association between later menarche and MD is not well understood. It is well established that adipose tissue deposition is needed for the onset of menarche and increased body adiposity is associated with earlier pubertal development [27]. In this study, we found that the magnitude of the association between age at menarche and PD was doubled without adjustment for BMI, whilst the DA-associated estimates remained similar. This finding highlights the importance of adjustment for BMI whenever estimating associations with PD. The estimates of association stratified by BMI were strongest (and largely driven by) women of average or below mean BMI (< = 25 kg/m^2^).

A recent review postulates that the timing of menarche, in terms of timing of availability of ovarian hormones, can impact breast morphology and, in turn, affect MD [11]. Women who experience a longer pubertal tempo, the time between the development of breast buds and menarche, have been shown to have increased dense area and increased breast cancer risk (independent of the age of onset of puberty) [11]. The review authors concluded that prolonged exposure of breast tissue to ovarian hormones could mediate these associations but further investigations are required.

The positive association of tall stature with breast cancer risk is not completely understood but the primary hypothesized mechanism is through the role of hormones and growth factors, particularly insulin-like growth factor-1 (IGF-1) via its stimulation of bone growth, the promotion of cell proliferation and inhibition of apoptosis [28, 29]. Genetic variants in the IGF pathway and circulating levels of IGF-1 have been associated with increased adult height [2, 30–32]. A Mendelian randomization study using height-associated genetic variants (including variants in the IGF pathway) suggested that adult height was not only a risk factor for breast cancer, but that the association was causal [2]. Circulating IGF-1 has also been independently associated with an increased risk of breast cancer, although with some inconsistent results regarding the effects of menopausal status and tumour subtype [31]. Further, increased levels of IGF-1 have also been associated with increased mammographic density [33, 34]. The increased production of cells in the breast due to increased IGF-1 is thought to lead to increased breast density and eventually to an increased risk of breast cancer [33, 34].

In this study, we found that increased adult height was positively associated with DA. The magnitude of the DA association was also largely independent of adjustment for BMI or age at menarche. Conversely, the association between height and PD is more complex, largely due to increased confounding and the large (expected) heterogeneity between PD and anthropometric measures across 22 international study populations. We found only marginal evidence of a positive association between PD and height but only without adjustment for BMI. Otherwise, PD was negatively associated with height. Taller women tended to have larger breasts (i.e. increased total breast area; data not shown) which may explain why increased height could be associated with lower PD.

The key strengths of this study were the large and ethnically and geographically diverse sample of women, and the comprehensive and harmonized data available across all studies. This also enabled reporting of both population- and group-specific associations, summarized using a meta-analytic approach, as well as overall associations estimated from the pooled individual-level data. The results were largely consistent using both approaches and for both mammographic measures (PD and DA), although the height–PD association depended upon the degree of body size adjustment. This suggests that the results are generalizable to women in populations worldwide.

There are limitations inherent in using existing data that were collected from multiple studies. In this case, this included some evidence of differences in associations with height when stratified by type of measurement (self-reported or measured), introducing a potential source of bias that needs to be taken into account when considering results. Previous studies that have shown the heritability of height vary depending on whether the height data were measured or self-reported [35]. Conversely, other studies have found high correlations between self-reported and measured height and weight in the same individuals, suggesting that the potential for bias may be minimized [36]. Unfortunately, we do not have self-reported and measured height and weight in the same individuals in the ICMD, which would be required to accurately assess the degree of bias. Adjustment for other anthropometric measures such as waist-to-hip ratio and percentage of body fat also warrant future investigation. A further complexity arises given measures of height vary with age. Adult height reflects the body’s linear growth, and maximum adult height is achieved in a woman’s adolescent or early adult years [37, 38]. However, height measured in later life, especially during post-menopausal ages, includes a degree of shrinkage. A further potential source of bias is therefore introduced depending on the age at which height was measured in each study.

## Conclusions

In summary, we have shown in one of the largest international studies to date that later age at menarche and increased adult height are both positively associated with measures of MD. The associations observed for height are in line with previous findings for associations with breast cancer risk, but those for menarche are in the opposite direction. These findings suggest that the association of age at menarche with breast cancer risk is not likely mediated through MD. Whether the well-established positive association of height with breast cancer risk is driven in whole or in part by elevated MD warrants further study. These results suggest a complex relationship between growth and development, MD and breast cancer risk.

## Supplementary Information

**Additional file 1. Tables S1 and S2:** Supplementary data providing results from sensitivity analyses of pooled models adjusted for a population-specific weight-for-height index instead of BMI

## Data Availability

ICMD data cannot be deposited publicly as these data originate from 27 research institutions across 22 countries with different legal and ethical frameworks. Researchers seeking the analysis data set for this work are able to apply to the Environment and Lifestyle Epidemiology Branch at the International Agency for Research on Cancer for access (email: env@iarc.fr).

## Abbreviations

BMI: Body mass index
DA: Dense area
ICMD: International Consortium of Mammographic Density
IGF: Insulin-like growth factor
IGF-1: Insulin-like growth factor-1
MD: Mammographic density
PD: Percent density
