# Supplementary material for: The association of age at menarche and adult height with mammographic density in the International Consortium of Mammographic Density
Source: Breast Cancer Res. 2022 Jul 14;24:49. doi: 10.1186/s13058-022-01545-9 (PMC9284807; doi:10.1186/s13058-022-01545-9)
Supplement: Supplementary file 1 — Additional file 1. Tables S1 and S2: Supplementary data providing results from sensitivity analyses of pooled models adjusted for a population-specific weight-for-height index instead of BMI [file 13058_2022_1545_MOESM1_ESM.pdf]

Supplementary Table S1: Correlation between height and BMI (weight/height^2) and the power coefficient for the best fitting weight/height^p relationship in each population group

Population Group	No. of women	Correlation between height and BMI (kg/m^2)	Power coefficient (k_2) in unit of weight for height, i.e. weight/height ^{k2}
UK-Eth-South Asian	135	-0.35	0.77
Turkey	395	-0.31	0.62
Malaysia-Indian	247	-0.30	0.82
Korea	389	-0.28	1.14
Australia-Italian	171	-0.25	0.96
US-USC-Asian	51	-0.22	1.05
UK-London-White	242	-0.22	0.92
South Africa	371	-0.21	1.09
UK-Eth Black	221	-0.20	1.17
US-MEC-Japanese	239	-0.20	1.00
Singapore-Indian	199	-0.20	1.19
Hong Kong	204	-0.19	1.26
Egypt	475	-0.18	1.12
US-NHS-White	397	-0.17	1.06
Israel-Arab	391	-0.17	1.18
Japan	384	-0.16	1.35
Spain	758	-0.16	1.28
UK-Eth-White	247	-0.15	1.20
UK-Age Trial-White	160	-0.15	1.30
Israel-Jewish	387	-0.14	1.30
Iran	392	-0.14	1.42
Netherlands	362	-0.13	1.48
US-Mayo-White	397	-0.13	1.24
US-USC-Black	112	-0.13	1.49
Singapore-Malay	197	-0.12	1.53
Australia-Greek	141	-0.11	1.53
Poland	396	-0.09	1.54
Canada	379	-0.09	1.53
Singapore-Chinese	196	-0.08	1.62
Australia-Australian	393	-0.08	1.63
US-USC-White	162	-0.08	1.54
US-MEC-White	153	-0.06	1.58
Norway	196	-0.06	1.78
Malaysia-Chinese	396	-0.05	1.82
Malaysia-Malay	217	-0.05	1.74
Mexico	147	-0.02	1.87
India	185	0.03	2.17
US-MEC-Hawaiian	142	0.03	2.16
Chile	187	0.09	2.39

Abbreviations: BMI: Body Mass Index; No.: number

Supplementary Table S2: Analyses using different methods to adjust (or not) for body fatness in the associations of menarche and height with percent density, dense area and breast area, with positive, null and inverse associations highlighted in green, blue and orange respectively

Outcome	Approach	Adjustment for body fatness		
		None	BMI	Excess weight for height[a]
		**Menarche (per 1 year increase)**		
PD	Meta	0.05 (0.03, 0.07)	0.02 (0.01, 0.04)	0.02 (0.01, 0.04)
PD	Pooled	0.06 (0.04, 0.08)	0.02 (0.01, 0.04)	0.02 (0.01, 0.04)
Dense area	Meta	0.04 (0.02, 0.06)	0.04 (0.02, 0.06)	0.04 (0.02, 0.06)
Dense area	Pooled	0.04 (0.02, 0.06)	0.03 (0.01, 0.04)	0.03 (0.01, 0.05)
Breast area	Meta	-0.06 (-0.09, -0.03)	0.02 (0.00, 0.04)	0.02 (0.00, 0.04)
Breast area	Pooled	-0.06 (-0.12, -0.06)	0.01 (-0.02, 0.03)	0.01 (-0.02, 0.03)
		**Height (per 10 cm increase)**		
PD	Meta	0.06 (0.01, 0.10)	-0.04 (-0.08, 0.00)	0.08 (0.04, 0.12)
PD	Pooled	0.05 (0.00, 0.09)	-0.06 (-0.10, -0.02)	0.07 (0.03, 0.11)
Dense area	Meta	0.08 (0.02, 0.13)	0.08 (0.02, 0.13)	0.08 (0.03, 0.13)
Dense area	Pooled	0.07 (0.01, 0.12)	0.06 (0.00, 0.11)	0.07 (0.01, 0.12)
Breast area	Meta	0.00 (-0.07, 0.07)	0.30 (0.25, 0.36)	-0.04 (-0.09, 0.02)
Breast area	Pooled	0.01 (-0.07, 0.08)	0.32 (0.27, 0.38)	-0.02 (-0.08, 0.04)

Abbreviations: BMI: Body Mass Index

[a] Residuals generated from regressing log(weight) on log(height) for each population group separately.
